# Stability of Poly[Ni(Salen)]-Based Electrodes in the Presence of Halide Impurities: Coordination and Redox Contributions

**DOI:** 10.3390/ijms27041816

**Published:** 2026-02-13

**Authors:** Daniil A. Lukyanov, Ulyana M. Rodionova, Peixia Yang, Ruopeng Li, Bo Wang, Oleg V. Levin, Dmitrii V. Anishchenko, Elena V. Alekseeva

**Affiliations:** 1 Institute of Chemistry, Saint-Petersburg University, St. Petersburg 199034, Russia; 2 MIIT Key Laboratory of Critical Materials, Technology for New Energy Conversion and Storage, School of Chemistry and Chemical Engineering, Harbin Institute of Technology, Harbin 150001, China

**Keywords:** nickel–Salen polymers, sterically hindered ligands, halide impurities, redox-active polymers, electrode stability, electrochemical degradation

## Abstract

The electrochemical stability of redox-active polymers based on Ni(II)–Salen complexes is of critical importance for their application as electrode materials for supercapacitors and lithium-ion batteries. This study presents a systematic analysis of the influence of fluoride, chloride, and bromide anions on the redox behavior of two polymeric films: *poly*[Ni(Salen)] and sterically protected *poly*[Ni(Saltmen)]. Using cyclic voltammetry (CV), electrochemical quartz crystal microbalance (EQCM), and X-ray photoelectron spectroscopy (XPS), we identify two distinct degradation mechanisms: (1) axial coordination of halide ions to the Ni(II) center followed by demetallation, which disrupts the conjugated system and reduces conductivity, and (2) oxidative halogenation of the ligand. In the presence of chloride ions, both *poly*[Ni(Salen)] and *poly*[Ni(Saltmen)] lose approximately 70% of their initial capacity over 50 cycles, indicating progressive electrochemical degradation. In contrast, both polymers demonstrate high electrochemical stability in bromide-containing electrolytes, retaining most of their capacity under identical conditions. Fluoride coordinates without compromising redox performance, serving as a model for electrochemically inert ligands. The results highlight the critical role of both electrolyte composition and ligand design in ensuring the long-term stability of nickel–Salen polymers in energy storage devices.

## 1. Introduction

The development of stable and efficient electrode materials is critical for advancing modern electrochemical energy storage systems, including Li-ion batteries and supercapacitors [1]. Electroactive polymers, such as polypyrrole, polyaniline, and polythiophene, have attracted attention due to their intrinsic conductivity and mechanical flexibility [2].

In the doped state, reported conductivities include from 17.1 to 60.9 S·cm^−1^ for one-dimensional polypyrrole nanostructures and 2–10 S·cm^−1^ for polyaniline in pressed-pellet form, while polar polythiophenes can reach conductivity up to 1173.9 S·cm^−1^ upon strong-acid vapor doping [3,4,5,6]. Mechanical robustness has been demonstrated in flexible conducting-polymer devices; for example, electropolymerized PEDOT:PSS supercapacitors operate at bending radius of 4.0–0.6 mm and retain ~94% of their capacitance after 1000 bending cycles [7].

These materials exhibit a unique combination of high electrical conductivity, reasonable stability, and favorable mechanical, optical, and electronic properties, which makes them highly promising for applications in sustainable energy technologies. Their high charge transfer rates and useful adhesion properties have long attracted scientific interest, even extending to their use as conductive binders in conventional lithium-ion batteries [8,9,10].

Among these materials, nickel complexes with polymeric Salen-type ligands have emerged as functional materials for a wide range of electrochemical applications [11]. Electropolymerized polymeric films based on nickel Schiff base complexes have been widely investigated as functional electrode materials due to their direct formation on conductive substrates, high interfacial adhesion, and stable redox activity. In particular, NiSalen-type polymer films have demonstrated pronounced pseudocapacitive behavior in supercapacitor electrodes with high capacity, depending on the complex structure and electrolyte composition [11,12,13,14]. Beyond supercapacitors, polymeric NiSalen derivatives have also been explored as cathode materials in rechargeable lithium-ion batteries, exhibiting stable cycling performance and efficient utilization of metal–ligand redox centers [15,16,17,18,19].

The electrochemical capacity of NiSalen-type polymer films strongly depends on the ligand structure and steric environment of the nickel coordination center. For simple Salen-based systems, relatively moderate capacities have been reported, for example 28 mAh·g^−1^ for unsubstituted poly[Ni(Salen)] measured by galvanostatic charge–discharge at 1 C, and 50 mAh·g^−1^ for poly[Ni(CH_3_Salen)] under similar conditions [16]. In contrast, structurally expanded ligands such as Saltmen lead to significantly enhanced pseudocapacitive performance, with poly[Ni(Saltmen)] exhibiting specific capacity around 70 F·g^−1^ from cyclic voltammetry [20]. Further structural modification also affects charge storage behavior. For instance, poly[Ni(CH_3_O-Saltmen)] films demonstrate maximum capacitances of approximately 130 F·g^−1^ [20]. Similarly, extended conjugation in poly[Ni(salphen)] results in specific capacitances around 85 F·g^−1^ measured by galvanostatic methods [21]. More recently, NiSalen-based polymer films have been implemented as functional switchable-resistance protective interlayers in lithium-ion battery systems to suppress overcharge-induced failure [16]. In addition, NiSalen-based polymer films have been successfully applied in photoelectrochemical and electrochromic systems [22,23], as well as in chemical and biosensors [24,25], further highlighting the multifunctional nature of these materials. These results demonstrate that the polymers represent a highly versatile and promising class of electroactive materials, since their molecular architecture can be systematically tailored to achieve targeted electrochemical properties through rational ligand design.

Despite exhibiting high cycling stability in rigorously anhydrous electrolytes [26,27,28], NiSalen-based polymers are often highly susceptible to moisture, such that even trace amounts of residual water in sealed electrochemical cells can result in significant performance degradation over extended cycling periods [29,30,31]. To mitigate this, structural modifications introducing steric groups—such as methyl—are helpful. Using a combination of cyclic voltammetry, EQCM, and XPS, the authors demonstrated that water coordination to the Ni(II) center leads to irreversible structural disruption and a gradual loss of electrochemical activity [30,31]. These works confirmed that steric hindrance around the metal center effectively suppresses water coordination and delays the onset of electrochemical degradation. This approach to structural stabilization has proven to be a viable strategy for enhancing the performance of electroactive polymers in humid environments.

In addition to water, halide anions may be considered as a relevant class of potential degradation agents for polymeric Ni complexes due to their nucleophilicity and coordination ability. They are frequently encountered in commercial electrolyte formulations as impurities or counter-ions in supporting salts such as LiPF_6_, LiBF_4_, and tetraalkylammonium salts. Furthermore, halides may originate from various stages of materials synthesis, including incomplete purification of starting reagents, residuals from synthetic precursors, or degradation of halogenated solvents under electrochemical conditions. According to our experience in the electrochemistry of NiSalen-type polymers, even at trace levels, halides can cause visible effects on the electrochemical properties of NiSalen-type polymers, leading to deterioration of electrode performance. However, the influence of the structural features of NiSalen complexes on the tolerance of the electroactive polymers to halide impurities in the electrolyte has not been discussed yet.

**Figure 1:**
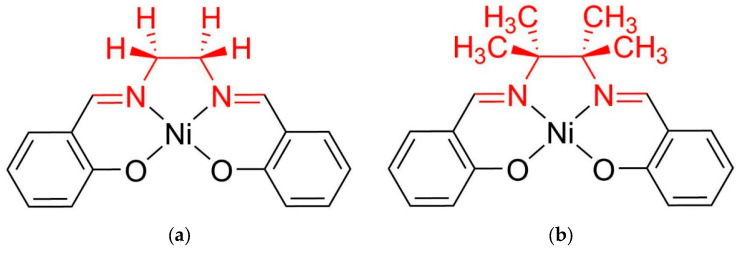
Structures of [Ni(Salen)] (**a**) and [Ni(Saltmen)] (**b**). The imine bridge fragments colored in red.

This study aims to fill this gap by evaluating the effects of fluoride, chloride, and bromide anions on the redox stability of two Ni-based polymers: one with minimal steric hindrance, *poly*[Ni(Salen)] (Figure 1a), and one with enhanced steric protection, *poly*[Ni(Saltmen)] (Figure 1b). Electrochemical stability is characterized by using CV, EQCM, and XPS. The results reveal distinct pathways of improving cycling stability of Ni-Salen based polymer electrodes by regulating the nature of the anion and the steric structure of the ligand, offering new insights into the design of stable redox-active polymers for electrochemical energy storage applications.

## 2. Results

The *poly*[Ni(Salen)] and *poly*[Ni(Saltmen)] complexes differ in the structure of the ligand backbone: the Saltmen ligand contains four methyl groups in the ethylenediamine bridge, resulting in increased steric hindrance around the coordination center and a modified electron density at the nickel atom. Thus, the changes in effect of the halide ions on the electrochemical properties moving from unhindered *poly*[Ni(Salen)] to highly hindered *poly*[Ni(Saltmen)] may provide information on the mechanisms of their interactions. We have implemented a series of halide ions, namely F^−^, Cl^−^ and Br^−^, which differ with their size, nucleophilicity/coordination ability and redox activity, to distinguish the possible mechanisms of interaction of the polymers with halide ions.

### 2.1. Effect of Fluoride Ions

**Figure 2:**
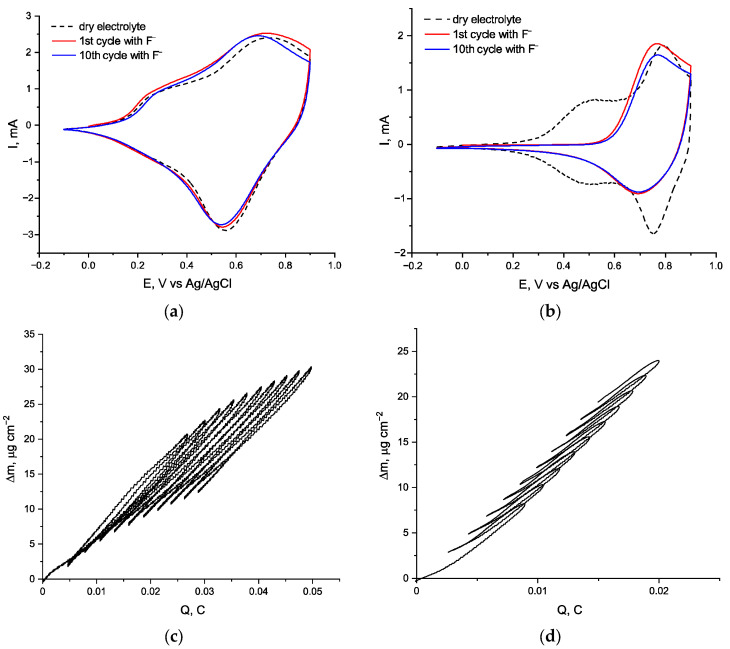
CV and corresponding EQCM responses of *poly*[Ni(Salen)] and *poly*[Ni(Saltmen)] in a dry acetonitrile electrolyte before and after fluoride addition: CV of *poly*[Ni(Salen)] (**a**) and *poly*[Ni(Saltmen)] (**b**); EQCM curves of *poly*[Ni(Salen)] (**c**) and *poly*[Ni(Saltmen)] (**d**). The supporting electrolyte was 0.1 M Et_4_NBF_4_ in CH_3_CN; fluoride was added as 1 mM Et_4_NF. CV scan rate is 50 mV·s^−1^. EQCM measurements were carried out using 5 MHz Ti/Pt-coated quartz crystals (active area 1.37 cm^2^); mass changes were calculated using the Sauerbrey equation.

Surprisingly, the addition of the F^−^ ion significantly affects the electroactivity of *poly*[Ni(Saltmen)] (Figure 2b), while *poly*[Ni(Salen)] remains nearly intact in the same conditions (Figure 2a). CV of *poly*[Ni(Saltmen)], which initially contains two distinct reversible peak pairs at 0.5 V and 0.77 V, loses the first peak immediately after the addition of fluoride, while the second peak pair becomes less reversible. At the same time, overall charge capacity of the film drops only by around 35%, which indicates that F^−^ ions cause redistribution of the peak currents rather than the degradation of electroactivity. Further cycling in fluoride-containing electrolytes only results in a minor degradation of the oxidation peak, while its reduction counterpart remains stable.

The mass of both films increases irreversibly cycle to cycle, which indicates the intake of the fluoride ions. However, *poly*[Ni(Salen)] gains 11 µg of excessive mass after 10 cycles (Figure 2c), which is about 50% of the mass change amplitude, and intake per cycle significantly decreases at this point, while *poly*[Ni(Saltmen)] gains 19.5 µg (Figure 2d) in the same conditions, which comprises more than 230% of the mass change amplitude, and the intake per cycle remains nearly the same with that observed at the first cycle. On the first slope of the inbound ionic flux upon oxidation, the *m*/*z* value comprises ca. 57 g·mol^−1^ for both *poly*[Ni(Salen)] and *poly*[Ni(Saltmen)], which is close to the mass of the F^−^•CH_3_CN particle. For the second slope of flux, the *m*/*z* value increases to 71 and 85 g·mol^−1^, respectively, which reflects that the BF_4_^−^ transport prevails on this stage. Upon reduction, exhaust BF_4_^−^ transport also prevails.

### 2.2. Effect of Chloride Ions

**Figure 3:**
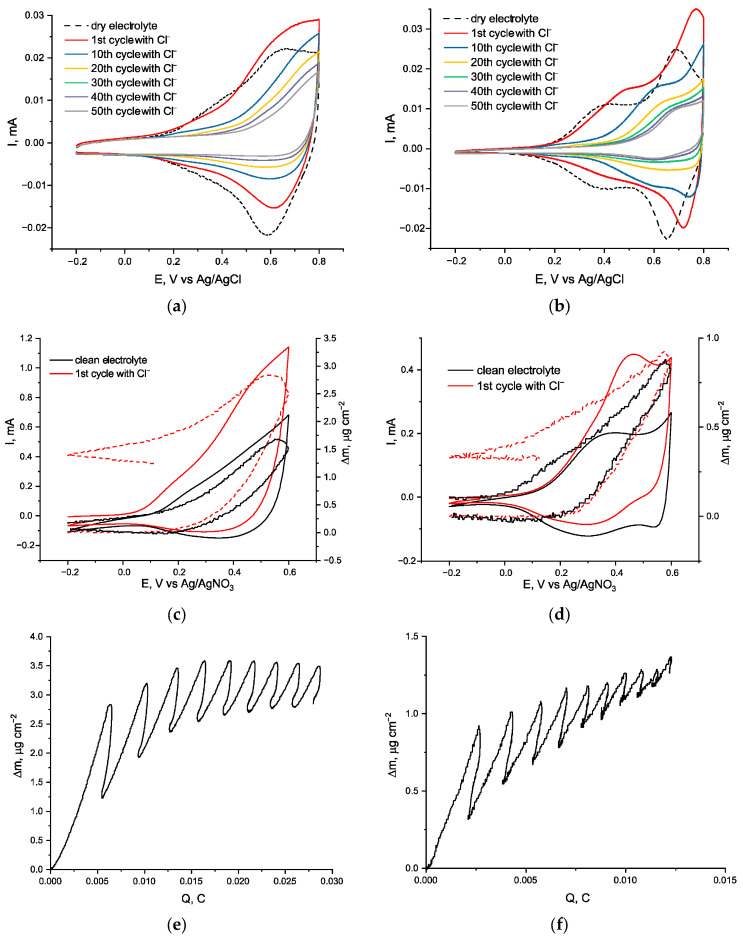
Electrochemical and EQCM characterization of *poly*[Ni(Salen)] and *poly*[Ni(Saltmen)] in chloride-containing electrolyte: CVs (**a**,**b**), mass–potential profiles (massopotentiograms) (**c**,**d**), and EQCM curves (**e**,**f**) for poly[Ni(Salen)] (**a**,**c**,**e**) and poly[Ni(Saltmen)] (**b**,**d**,**f**) after addition of chloride. Measurements were performed in 0.1 M Et_4_NBF_4_/CH_3_CN (dry), with chloride introduced as 1 mM Et_4_NCl. CV scan rate is 50 mV·s^−1^. EQCM was recorded on 5 MHz Ti/Pt-coated quartz crystals (active area 1.37 cm^2^); mass changes were calculated using the Sauerbrey equation. Dashed lines correspond to cycling in the supporting electrolyte without halide addition.

The addition of Cl^−^ had a pronounced effect on the electrochemical behavior of both polymers over the course of 50 cycles (Figure 3a,b). Inherent redox activity of both polymers gradually degrades with comparable rates until the disappearance of the reduction peaks, leaving only an irreversible shoulder of Cl^−^ oxidation. However, in case of *poly*[Ni(Saltmen)], some minor polymer-based electroactivity remains after 50 cycles. Similarly to for fluoride-promoted degradation, the low-potential peak pair disappears rapidly, while the high-potential peak remains for much longer.

In the case of *poly*[Ni(Salen)] (Figure 3a), the capacity of the film decreased by 65% upon addition of Et_4_NCl (Cl^−^). In the case of the addition of Cl^−^ to electrolyte *poly*[Ni(Saltmen)], it exhibited around 70% loss in capacity (Figure 3b).

After adding Cl^−^ ions, the oxidation ion flux for both polymers becomes linear with a single slope of 34 g·mol^−1^, in contrast to the bilinear curves seen in the control experiment without an additive, suggesting [31] a single dominant ion transport process with the ion flux of 34 g·mol^−1^, which corresponds to the Cl^−^ ion (Figure 3e,f). Although the *m*/*z* of the reduction mass flux for both polymers is much higher than *m*/*z* of the oxidation mass flux and correspond to the exit of the BF_4_^−^ anions, both polymers irreversibly gain mass during each CV cycle, which indicates that the Cl^−^ ions do not only substitute the BF_4_^−^ as a dopant, but irreversibly incorporate in the structure of the polymers. In the case of *poly*[Ni(Salen)], the mass gain stops after five cycles and turns to a slight decrease in the subsequent cycles, while *poly*[Ni(Saltmen)] gains mass with nearly constant rate all 10 cycles, which indicates lower rate of degradation.

### 2.3. Effect of Bromide Ions

**Figure 4:**
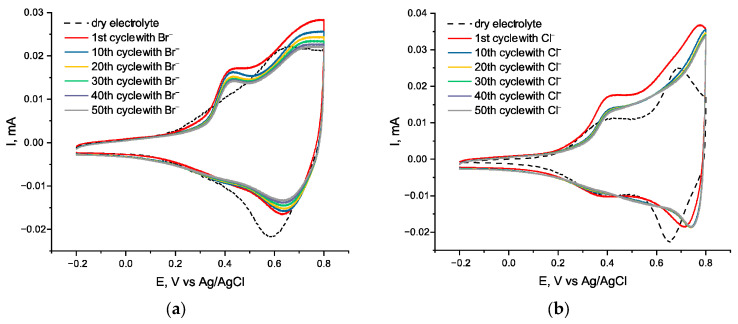

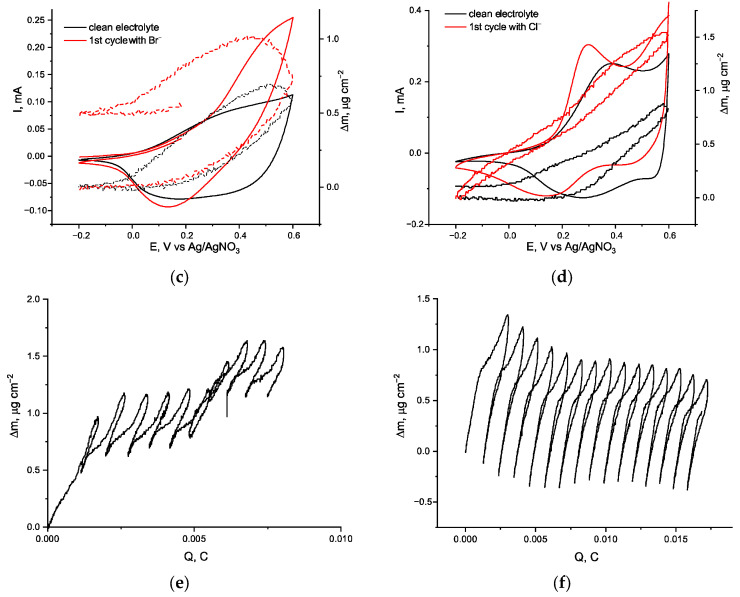
Electrochemical and EQCM characterization of *poly*[Ni(Salen)] and *poly*[Ni(Saltmen)] in chloride-containing electrolytes: CVs (**a**,**b**), mass–potential profiles (massopotentiograms) (**c**,**d**), and EQCM curves (**e**,**f**) for poly[Ni(Salen)] (**a**,**c**,**e**) and poly[Ni(Saltmen)] (**b**,**d**,**f**) after addition of bromide. Measurements were performed in 0.1 M Et_4_NBF_4_/CH_3_CN (dry), with bromide introduced as 1 mM Et_4_NBr. CV scan rate is 50 mV·s^−1^. EQCM was recorded on 5 MHz Ti/Pt-coated quartz crystals (active area 1.37 cm^2^); mass changes were calculated using the Sauerbrey equation. Dashed lines correspond to cycling in the supporting electrolyte without halide addition.

Upon the addition of Br^−^ ions, two peak pairs emerge at 0.4 and ca. 0.8 V, corresponding to the two-step oxidation of Br^−^ to Br_2_ via an intermediate Br_3_^−^. Since the electroactivity of bromide ions massively overlap the peaks of the polymers, it is impossible to make a conclusion regarding their electrochemical stability, but since the overall CV curves show only minor shrinkage after 50 cycles, the films are likely to be nearly stable in the presence of bromide. However, the stability of *poly*[Ni(Salen)] (Figure 4a) seems to be slightly worse than *poly*[Ni(Saltmen)] (Figure 4b).

During EQCM, *poly*[Ni(Saltmen)] (Figure 4d,f) exhibits good behavior with slow cycle-to-cycle mass decrease, which may be due to the ion exchange of BF_4_^−^ to Br^−^, and stable mass amplitude. The ion flux is approximately 78 g·mol^−1^ on the first slope, which corresponds to the inbound flux of Br^−^ ions, and 27 on the second slope, which can be explained by an inbound flux of Br^−^ ions, accompanied by an exhaust flux of neutral Br_2_. The EQCM of *poly*[Ni(Salen)] (Figure 4c,e) is much more complicated, but apparently, the *poly*[Ni(Salen)] film gains significant mass from cycle to cycle, showing that bromide incorporates in the structure of this polymer to some extent, which agrees with its lower electrochemical stability.

To further assess the morphological and elemental stability of the polymer films after electrochemical cycling in chloride-containing electrolytes, SEM imaging and EDX mapping were performed for both poly[Ni(SalEn)] and poly[Ni(Saltmen)] electrodes (Figures S1 and S2). The SEM images revealed that the morphology of the films was preserved after 50 CV cycles, indicating the absence of mechanical degradation or delamination. Elemental mapping of nickel by EDX confirmed that the surface distribution of Ni remained largely uniform, supporting the structural stability of the films.

However, semi-quantitative surface analysis indicated a detectable change in composition: both systems showed the presence of chlorine on the surface after cycling, suggesting that Cl^−^ anions are coordinated to the nickel centers or embedded in the film matrix. The observed behavior was consistent for both poly[Ni(SalEn)] and poly[Ni(Saltmen)] films, demonstrating that although the polymer frameworks remain morphologically stable, the surface undergoes chemical transformation due to anion uptake. These findings are in agreement with the hypothesis that a loss of electrochemical activity is not driven by mechanical damage, but rather by changes in the redox conductivity of the conjugated system induced by halide coordination.

### 2.4. XPS Analysis

To confirm the incorporation of external ligands into the polymer films, X-ray photoelectron spectroscopy of the polymeric films before and after 50 cycles in halide-containing electrolytes was performed (Figure S3). The Ni 2p, C 1s, N 1s and F 1p or Cl 2p peaks were compared and analyzed.

**Figure 5:**
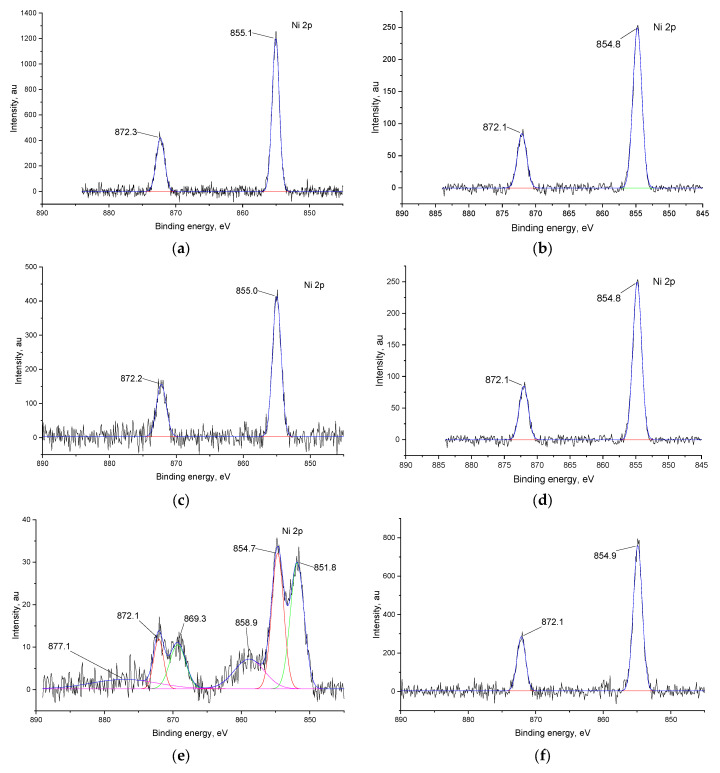
Ni 2p XPS spectra of *poly*[Ni(SalEn)] before (**a**) and after 50 CV cycles in electrolyte with addition of 1 mM Et_4_NF (**c**) and 1 mM Et_4_NCl (**e**); spectra of *poly*[Ni(SaltmEn)] before (**b**) and after 50 CV cycles in electrolyte with addition of 1 mM Et_4_NF (**d**) and 1 mM Et_4_NCl (**f**). In each panel, the black line represents the experimental spectrum, the blue line represents the overall fitted spectrum, to red, green and violet lines represent the fitted components of the Ni 2p_3_/_2_ and Ni 2p_1_/_2_.

In the pristine state, before the addition of halide ions, the Ni 2p_3/2_ spectra of both polymers (Figure 5a,b) display characteristic peaks at ca. 855 and 872 eV. These peaks correspond to Ni(II) in a square-planar N,N,O,O-coordination environment, which is typical for nickel complexes with Salen-type ligands [30]. The same pattern is observed for all films with halide ions (Figure 5c,d,f), except the ion of *poly*[Ni(Salen)] after the addition of Cl^−^ ions (Figure 5e). In this case, there are two additional peaks at 851.8 and 858.9 eV. The first peak corresponds to the metallic Ni, ref. [32], while the second one can be attributed to nickel oxide or chloride [33].

**Figure 6:**
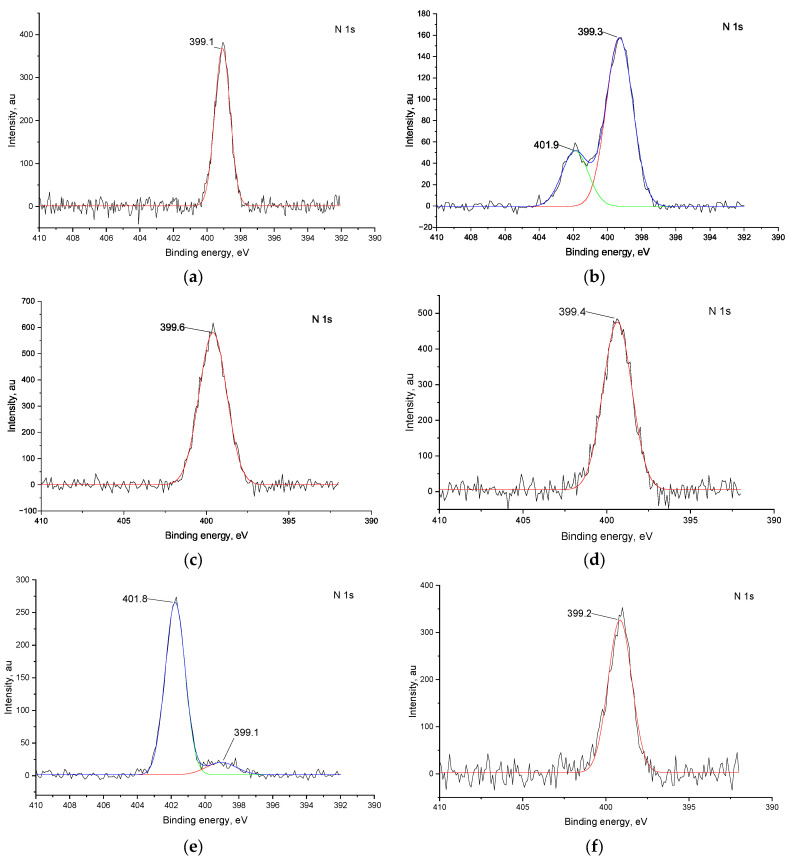
N 1s XPS spectra of *poly*[Ni(SalEn)] before (**a**) and after 50 CV cycles in electrolyte with addition of 1 mM Et_4_NF (**c**) and 1 mM Et_4_NCl (**e**); spectra of *poly*[Ni(SaltmEn)] before (**b**) and after 50 CV cycles in electrolyte with addition of 1 mM Et_4_NF (**d**) and 1 mM Et_4_NCl (**f**). In panel (**b**,**e**), the black line represents the experimental spectrum, the blue line represents the overall fitted spectrum, the red and green lines represent the fitted components of the N 1s. In panel (**a**,**c**,**d**,**f**) the black line represents the experimental spectrum, the red line represents the fitted components of the N 1s.

N 1s spectra (Figure 6) of the pristine films contain one peak at ca. 399 eV, corresponding to the imine nitrogen, coordinated to the Ni atom, ref. [34], while there is an additional peak at ca. 402 eV due to the presence of the Et_4_N^+^ ions from the electrolyte [34]. The only sample that shows changes after CV is again *poly*[Ni(Salen)] after Cl^−^ ions. The initial peak at ca. 399 eV nearly disappears, replaced by a new peak at ca. 402 eV, which can be attributed to the Et_4_N^+^ ions.

**Figure 7:**
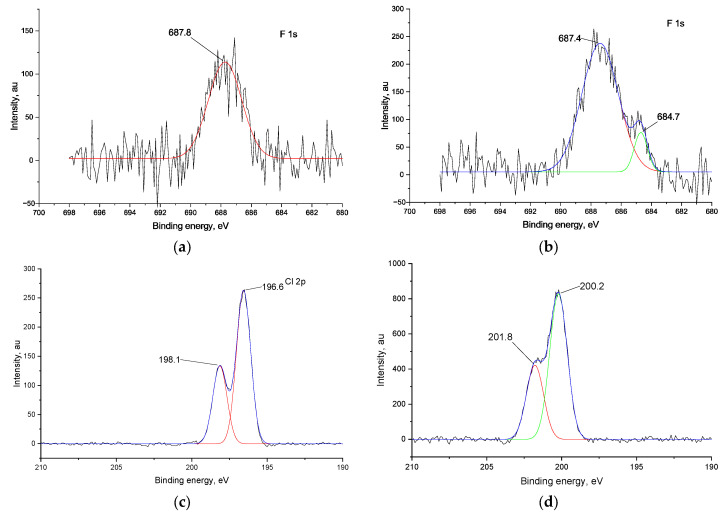
F 1s XPS spectra of polymeric films after CV in halide-containing electrolytes (**a**) *poly*[Ni(Salen)] and (**b**) *poly*[Ni(Saltmen)]; Cl_2p_ spectra of polymeric films after CV in halide-containing electrolytes (**c**) *poly*[Ni(Salen)] and (**d**) *poly*[Ni(Saltmen)]. In each panel, the black line represents the experimental spectrum, the blue line represents the overall fitted spectrum, red and green lines represent the fitted components of the F 1s and Cl 2p.

After CV in fluoride-containing electrolytes, a peak at ca. 687.5 eV emerges in the F 1s spectra of both films (Figure 7b), which is attributed to the fluorination of the aromatic rings of the ligand [35]. In the same manner, cycling of *poly*[Ni(Saltmen)] in a chloride-containing electrolyte results in the chlorination of the aromatic rings of the ligand, since the Cl 2p peak at 200.2 eV is typical for chlorobenzenes [36] (Figure 7d). In the case of *poly*[Ni(Salen)], a peak at 196.6 eV emerges after CV with Cl^−^ ions, which can be attributed to the Cl^−^ ions [37] (Figure 7c).

**Figure 8:**
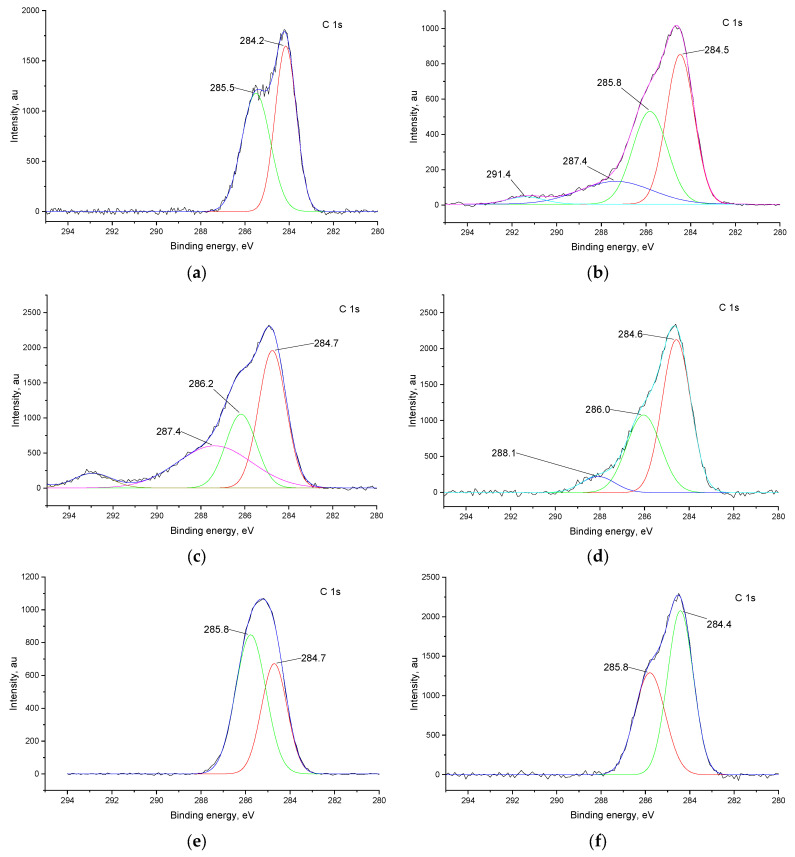
C 1s XPS spectra of *poly*[Ni(SalEn)] before (**a**) and after 50 CV cycles in electrolyte with addition of 1 mM Et_4_NF (**c**) and 1 mM Et_4_NCl (**e**); spectra of *poly*[Ni(Saltmen)] before (**b**) and after 50 CV cycles in electrolyte with addition of 1 mM Et_4_NF (**d**) and 1 mM Et_4_NCl (**f**). In each panel, the black line represents the experimental spectrum, the blue line represents the overall fitted spectrum, to red and green lines represent the fitted components of the C 1s.

In the C 1s spectra of all films (Figure 8), there are two predominant peaks at ca. 284.5 and 286.0 eV, which correspond to the carbon atoms of the ligand backbone. In addition, in F^−^-treated samples, a new peak emerges at 287–288 eV, which may be attributed to the fluorinated carbon atoms. In contrast, no additional peaks are present in the spectra after Cl^−^ treatment, which is due to the fact that the peaks of chlorinated carbons usually situate near 286 eV and overlap with existing peaks.

Concluding the results of XPS, both polymers undergo fluorination of the ligand upon CV with F^−^ ions. With Cl^−^ ions, *poly*[Ni(Saltmen)] undergoes chlorination as well, while *poly*[Ni(Salen)] suffers destruction of the complex, while in other cases the coordination cavity remains intact.

## 3. Discussion

A comparative analysis of the obtained CV and EQCM data shows the following trend: relative electrochemical stability of *poly*[Ni(Saltmen)] to *poly*[Ni(Salen)] increases in the row F^−^ < Cl^−^ < Br^−^, or, in other words, small, hard and nucleophilic anions better attack *poly*[Ni(Saltmen)], while large, soft and weekly nucleophilic anions better attack *poly*[Ni(Salen)]. Such an effect of the size of the attacking particle can be easily explained by the higher steric hindrance of *poly*[Ni(Saltmen)], but the opposite effect observed in the case of *poly*[Ni(Salen)] needs particular consideration regarding the possibility of interaction mechanisms.

**Figure 9:**
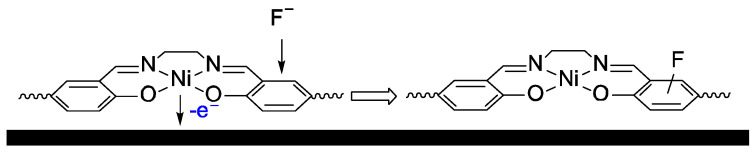
Schematic representation of degradation processes occurring in both *poly*[Ni(Salen)] and *poly*[Ni(Saltmen)] polymer films upon interaction with fluoride-containing electrolyte.

According to the EQCM, both polymers uptake the fluoride anion in oxidized form. However, this process does not affect the electroactivity of *poly*[Ni(Salen)], while the low-potential peak of *poly*[Ni(Saltmen)] totally disappears after the first cycle. Such behavior may be explained by the fluorination of the ligand (Figure 9), because F^−^ is a much stronger nucleophile in aprotic and moderately polar CH_3_CN, in comparison with other halide ions. F^−^ is expected to fluorinate the benzene ring of the radical cationic species localized on the Salen ligands after oxidation, since fluorination of the electrochemically generated radical cationic species is widely described in the literature [38]. In contrast to the coordination on the Ni atom, which disables the conductivity and electroactivity of the polymer, benzene ring fluorination does not interrupt the conductivity pathway along the polymer chain. In case of *poly*[Ni(Saltmen)], the fluorination of the benzene rings may cause the distortion of the complex plane due to the repulsion of F^−^ substituents with methyl groups of the imine bridge, and thus prevent the polaron delocalization, which is known to be responsible for the low-potential peak of *poly*[Ni(Saltmen)] [39].

**Figure 10:**
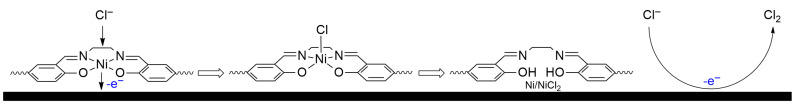
Schematic representation of degradation processes occurring in *poly*[Ni(Salen)] polymer films upon interaction with chloride-containing electrolyte.

Upon oxidation of *poly*[Ni(Salen)] in a chloride-containing electrolyte, chloride anions readily coordinate to the oxidized nickel centers within the polymer’s backbone (Figure 10). This axial coordination leads to a marked decrease in redox activity, analogous to the demetallation of the NiSalen complex, as previously observed in aqueous environments. At the same time, the Ni atom in the *poly*[Ni(Saltmen)] complex is sufficiently hindered by four bulky methyl substituents on both sides of the complex plane. Instead, oxidative chlorination of the aryl rings of the ligand occurs, analogous to a previously discussed fluorination reaction. In parallel, chloride ions may also undergo electrochemical oxidation at the electrode surface, producing molecular chlorine, which subsequently evolves from the system. This additional process contributes to the CV of both *poly*[Ni(Salen)] and *poly*[Ni(Saltmen)] films.

The bromide ion, due to its lower oxidation potential, undergoes electrochemical oxidation via a two-step process involving the intermediate formation of tribromide (Br_3_^−^). This behavior is evidenced by the appearance of two well-defined bromide-related peaks in the voltammogram, while the polymer retains its intrinsic redox activity [40]. *Poly*[Ni(Salen)] still suffers slow degradation, which is apparently caused by the coordination of the bromide ion to the Ni center, while *poly*[Ni(Saltmen)] is completely resistant to this process.

It should be noted that in electrolytes containing Cl^−^ and especially Br^−^, partial oxidation of halide anions may occur in the same potential window as the redox transitions of the Ni–Salen polymer. This overlap could potentially complicate the direct attribution of current responses to polymer redox activity. However, control experiments with blank electrodes and the absence of irreversible oxidative signals in pristine films suggest that the majority of the changes observed during cycling are due to polymer degradation rather than halide oxidation.

While the observed mass changes in EQCM experiments may partly originate from swelling or partial entrapment of electrolyte species, the complementary XPS and EDX data confirm the presence of halide ions on the polymer surface after cycling. This supports the interpretation that specific halide coordination contributes to the observed mass increase and redox behavior.

To place the obtained results into a broader research context and to better rationalize the observed degradation trends, we summarized and compared the electrochemical performance of the present *poly*[Ni(Salen)] and *poly*[Ni(Saltmen)] films with the representative literature on structurally related nickel–Salen and nickel–salphen polymers (Table 1). Due to the variety of nickel–Salen-type polymer structures and their applications, the collected data span different device concepts (supercapacitors vs. Li-ion batteries), electrolytes (carbonate- and acetonitrile-based aprotic media, as well as alkaline aqueous electrolytes), film architectures (pristine polymers vs. carbon-based composites), and electrochemical protocols (CV, GCD, and EIS/spectroelectrochemistry). Therefore, Table 1 is not intended to provide a strictly quantitative benchmark, but rather to illustrate the diversity of reported performances and the factors most frequently discussed in the literature.

Importantly, the cycling stability reported for Ni–Salen-type polymer electrodes shows pronounced scatter across studies, even for closely related ligand frameworks. Depending on the electrolyte formulation, testing protocol, and film morphology, retention values range from nearly quantitative stability in rigorously controlled “dry” aprotic systems to rapid losses in the presence of nucleophilic or redox-active species (e.g., H_2_O and halides). Notably, although ligand design and steric shielding are often emphasized as key stabilization strategies, systematic investigations that isolate the role of electrolyte composition—particularly the impact of trace impurities—remain limited. The broad dispersion of stability metrics reported in the literature is consistent with the notion that minor contaminants can disproportionately influence degradation pathways and may partially explain why comparable Ni–Salen polymer electrodes sometimes exhibit markedly different durability in nominally similar experiments.

In this context, the present work provides a controlled and mechanistically oriented assessment of electrolyte impurity effects by deliberately introducing F^−^, Cl^−^, and Br^−^ (1 mM) into a well-defined acetonitrile-based supporting electrolyte and interrogating the films by complementary CV, EQCM, and XPS/EDX analyses. Our results show that chloride causes substantial pseudocapacitance loss for both protected and unprotected polymers over prolonged cycling, whereas the bromide-containing electrolyte preserves the redox response to a much greater extent, particularly for the sterically protected poly[Ni(Saltmen)]. These findings emphasize that the apparent stability of Ni–Salen polymers cannot be interpreted independently of electrolyte purity and impurity chemistry, and rational ligand steric design should be considered together with electrolyte impurity control when targeting durable polymer electrodes for practical electrochemical energy storage environments.

## 4. Materials and Methods

### 4.1. Materials and Synthesis

All reagents were purchased from commercial suppliers and used as received unless otherwise stated. Anhydrous acetonitrile (99.9%) was further dried over activated 3 Å molecular sieves for at least 20 days prior to use. The final water content was controlled by Karl Fischer titration and did not exceed 10 ppm. Supporting electrolytes were prepared by dissolving in a solution containing 0.1 M Et_4_NBF_4_ in CH_3_CN. Halide additives (tetraethylammonium chloride (Et_4_NCl), tetraethylammonium bromide (Et_4_NBr), or tetraethylammonium fluoride (Et_4_NF)) were introduced at concentrations of 1 mM (10^−3^ M). Nickel(II) complexes of Schiff base ligands (Figure 1) were synthesized as previously described [43].

The ligands were obtained via condensation of the corresponding salicylaldehyde derivatives with ethylenediamine or 2,2,3,3-tetramethylethylenediamine under reflux in ethanol. The resulting complexes were purified by recrystallization.

### 4.2. Electrochemical Characterization

Electrochemical measurements were performed using Autolab PGSTAT30 and PGSTAT302N potentiostats (Metrohm Autolab B.V., Utrecht, The Netherlands). A conventional three-electrode cell was employed with a glassy carbon disk (0.07 cm^2^) as the working electrode, a platinum foil as the counter electrode, and a Ag|Ag^+^ reference electrode (acetonitrile solution of 0.1 M Et_4_NBF_4_ + 5 mM AgNO_3_). All potentials are reported versus this pseudo-reference electrode. Polymer films were deposited electrochemically on glassy carbon or platinum substrates from 1 mM monomer solutions in acetonitrile containing 0.1 M Et_4_NBF_4_. Potentiostatic electropolymerization was carried out at 0.8 V vs. Ag|Ag^+^ until a charge density of 0.04 C·cm^−2^ was reached. To assess the influence of polymer film thickness on degradation behavior, all samples in this study were synthesized using the same electropolymerization protocol to ensure comparable thicknesses. This approach allowed us to isolate the effect of halide coordination on electrochemical stability without introducing variability from morphological or thickness-related gradients.

Cyclic voltammetry was conducted in the potential window from –0.2 V to +0.6 V (or +0.8 V for Saltmen-type films) at a scan rate of 50 mV·s^−1^. After electropolymerizing, the films were first tested in a pure supporting electrolyte for 5 cycles, followed by an additional 50 scans after the introduction of halide anions. For each condition, at least three independent electrodes were tested to ensure reproducibility.

EQCM measurements were performed using a QCM200 quartz microbalance system (Stanford Research Systems, Sunnyvale, CA, USA) equipped with 5 MHz AT-cut quartz crystals coated with Ti/Pt (active area: 1.37 cm^2^). The film was electropolymerized under potentiostatic conditions at 0.8 V vs. Ag|Ag^+^ until a charge density of 0.95 C·cm^−2^. After electropolymerizing, the first 5 cycles were tested in a pure supporting electrolyte and then 50 scans were conducted after the introduction of halide anions. Mass changes were monitored during potential cycling under identical electrochemical conditions. Ion flux *m*/*z* values were calculated using the Sauerbrey equation [44].

### 4.3. Physicochemical Characterization

X-ray photoelectron spectroscopy was performed using a Thermo Fisher Scientific Escalab 250Xi (Waltham, MA, USA) instrument equipped with a non-monochromatized Al Kα X-ray source (1486.6 eV). Spectra were collected with a pass energy of 20 eV and an energy resolution better than 0.8 eV. The pressure in the analysis chamber was maintained below 10^−8^ mbar during measurements.

Polymer films for XPS analysis were electropolymerized on platinum foil substrates using the same conditions as for electrochemical experiments. Prior to XPS measurements, the samples were subjected to 50 CV cycles in a halide-containing electrolyte to simulate typical degradation conditions.

## 5. Conclusions

As a result of the comprehensive analysis of the influence of halide anions (Cl^−^, Br^−^, and F^−^) on the electrochemical stability of nickel–Schiff base polymer films, we established that the sterically hindered complex *poly*[Ni(Saltmen)] is vulnerable to strong nucleophiles like F^−^ through the oxidative ligand halogenation, while the less hindered *poly*[Ni(Salen)] suffers from the destruction of the coordination cavity by Cl^−^ ions. We found the bromide ion to be the most harmless halide ion due to its bulkiness and low oxidation potential, although the steric protection increased the stability of the complexes against the attack of this ion. We identified two principal degradation mechanisms: (1) axial coordination of external ligands, which disrupts the conjugated system and reduces charge transport, and (2) ligand-centered halogenation of the ligand. The interplay between these factors governs the long-term stability or degradation of the electroactive polymer films.

These findings underscore the critical importance of both electrolyte composition and ligand molecular architecture in ensuring the durability of Ni-based redox polymers. Strategies focused on metal center shielding and the exclusion of electrochemically active impurities offer a promising route toward the rational design of stable materials for supercapacitor applications.

## Figures and Tables

**Table 1:** Comparison of electrochemical behavior of Ni–Salen-derived polymeric films.

Polymer Composition	Steric Protection	Electrolyte (Conditions)	Cycling Performance	Reference
*poly*[Ni(Salen)]	none	1 M LiPF_6_ in EC/DEC	~95% capacity retained after 100 cycles	[16]
*poly*[Ni(CH_3_Salen)]	partial	1 M LiPF_6_ in EC/DEC	~90% capacity retention after 100 cycles	[16]
*poly*[Ni(Saltmen)]	methyl groups in imine bridge	0.1 M Et_4_NBF_4_ + 1% H_2_O in CH_3_CN	~98% capacity retention after 50 cycles	[31]
*poly*[Ni(salphen)@rGO/MWCNT composite]	none; composite with reduced graphene oxide/multiwall carbon nanotubes	Et_4_NBF_4_/CH_3_CN	~90% pseudocapacitance retained after 1000 cycles	[12]
meso-Ni-SaldMe-3dMe	partial	0.1 M (TBA)PF6, in propylene carbonate	Retain ~96% of their normalized charge after 200 consecutive CV cycles	[41]
RGO/*poly*[meso-Ni(II)-SaldMe]	partial; composite with reduced graphene oxide	0.1 M (TBA)PF6, in propylene carbonate	Retain ~90% of their normalized charge after 200 consecutive CV cycles	[42]
*poly*[Ni(Salen)]	none	0.1 M Et_4_NBF_4_ in CH_3_CN	Stable redox response in clean electrolyte	This work
*poly*[Ni(Saltmen)]	methyl groups in imine bridge	0.1 M Et_4_NBF_4_ in CH_3_CN	Stable redox response in clean electrolyte	This work
*poly*[Ni(Salen)]	none	0.1 M Et_4_NBF_4_ + 1 mM Et_4_NCl in CH_3_CN	~70% capacity loss over 50 cycles (≈30% retained)	This work
*poly*[Ni(Saltmen)]	partial	0.1 M Et_4_NBF_4_ + 1 mM Et_4_NCl in CH_3_CN	~70% capacity loss over 50 cycles (≈30% retained)	This work

## Data Availability

The original contributions presented in this study are included in the article/Supplementary Materials. Further inquiries can be directed to the corresponding author.

## Supplementary Materials

The following supporting information can be downloaded at: https://www.mdpi.com/article/10.3390/ijms27041816/s1.

## Abbreviations

The following abbreviations are used in this manuscript:

CV	Cyclic voltammetry
EQCM	Electrochemical quartz crystal microbalance
XPS	X-ray photoelectron spectroscopy
