# Supplementary material for: Stability of Poly[Ni(Salen)]-Based Electrodes in the Presence of Halide Impurities: Coordination and Redox Contributions
Source: Int J Mol Sci. 2026 Feb 13;27(4):1816. doi: 10.3390/ijms27041816 (PMC12941359; doi:10.3390/ijms27041816)
Supplement: Supplementary file 1 [file ijms-27-01816-s001.zip › ijms-4094522-supplementary.pdf]

–*Supplementary information*

Stability of Poly[Ni(Salen)]-based electrodes in the presence of Halide Impurities: Coordination and Redox Contributions

Daniil A. Lukyanov [1], Ulyana M. Rodionova [1], Peixia Yang [2], Ruopeng Li [2], Bo Wang [2], Oleg V. Levin [1], Dmitrii V. Anishchenko [1] and Elena V. Alekseeva [1,*]

[1] Institute of Chemistry, Saint-Petersburg University, 199034 St. Petersburg, Russia
[2] Harbin Institute of Technology, Harbin 150001, China
* Correspondence: e.v.alekseeva@spbu.ru

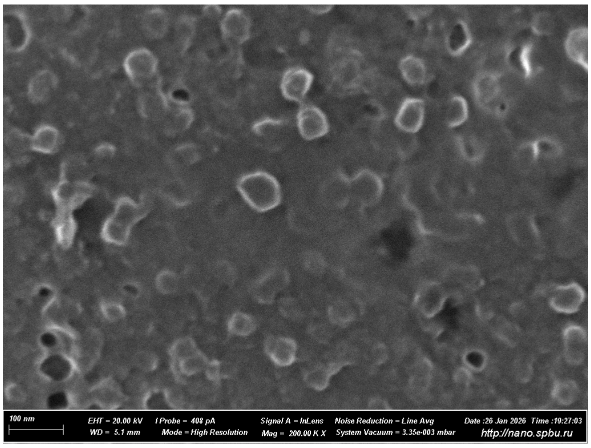

(a)

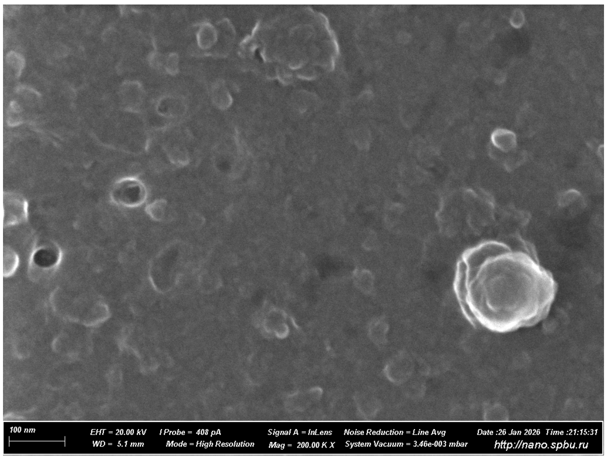

(b)

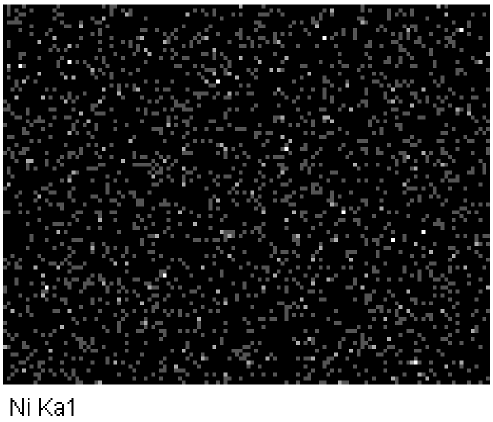

(c)

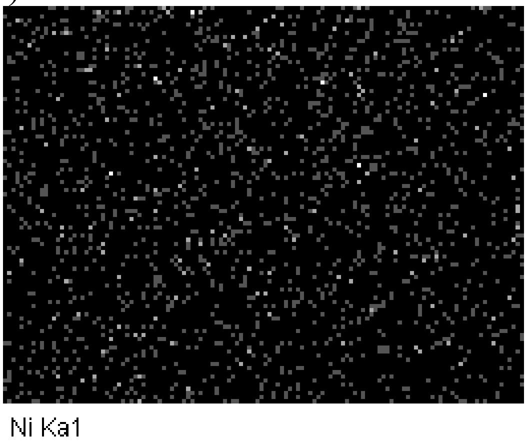

(d)

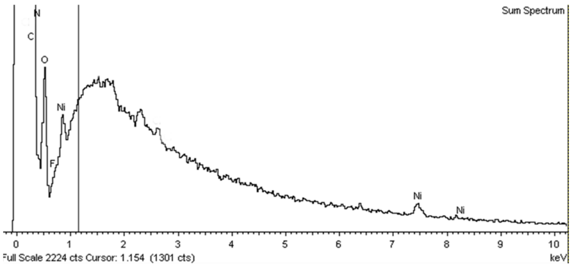

(e)

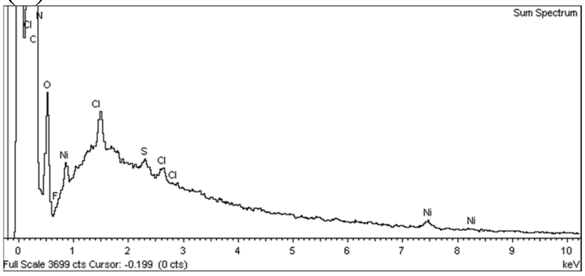

(f)

Figure S1. SEM images of poly[Ni(SalEn)] films before (a) and after (b) 50 CV cycles in the presence of 1 mM Et_4NCl; corresponding Ni EDX elemental maps before (c) and after cycling (d); and surface elemental composition derived from EDX analysis before (e) and after (f) cycling.

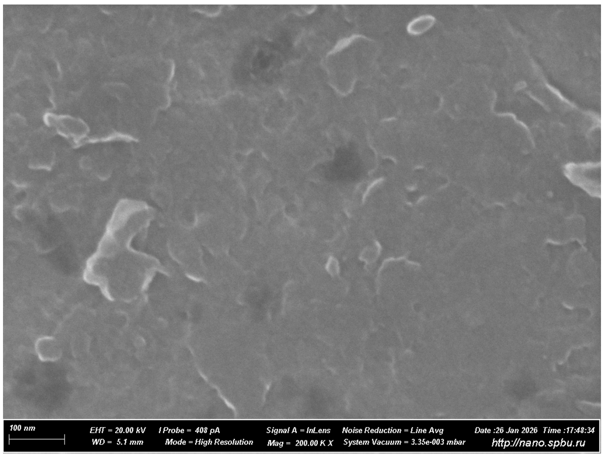

(a)

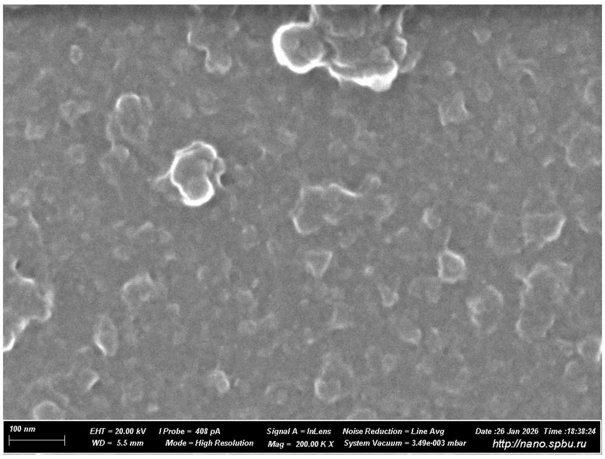

(b)

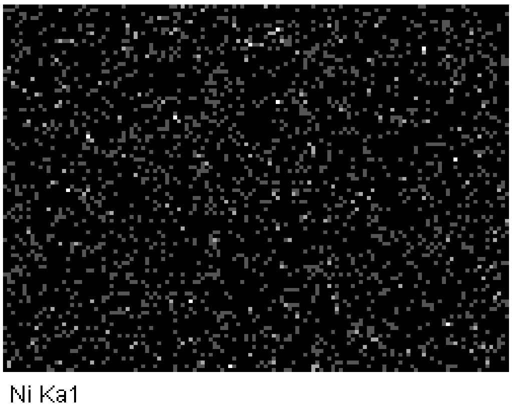

(c)

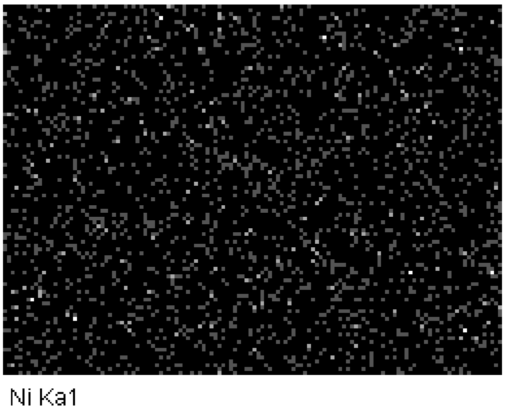

(d)

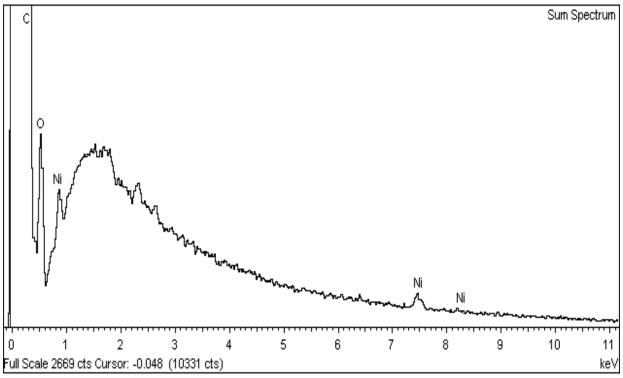

(e)

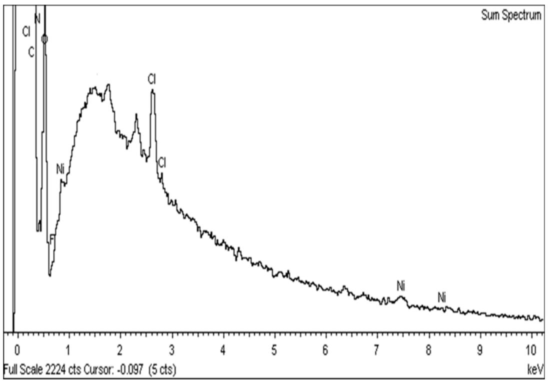

(f)

Figure S2. SEM of *poly*[Ni(Saltmen)] before (**a**) and after 50 CV cycles in electrolyte with addition of 1 mM Et_4NCl (b) EDX картирование по никелю before (c) and after 50 CV cycles in electrolyte with addition of 1 mM Et_4NCl (d) состав поверхности before (e) and after 50 CV cycles in electrolyte with addition of 1 mM Et_4NCl (f).

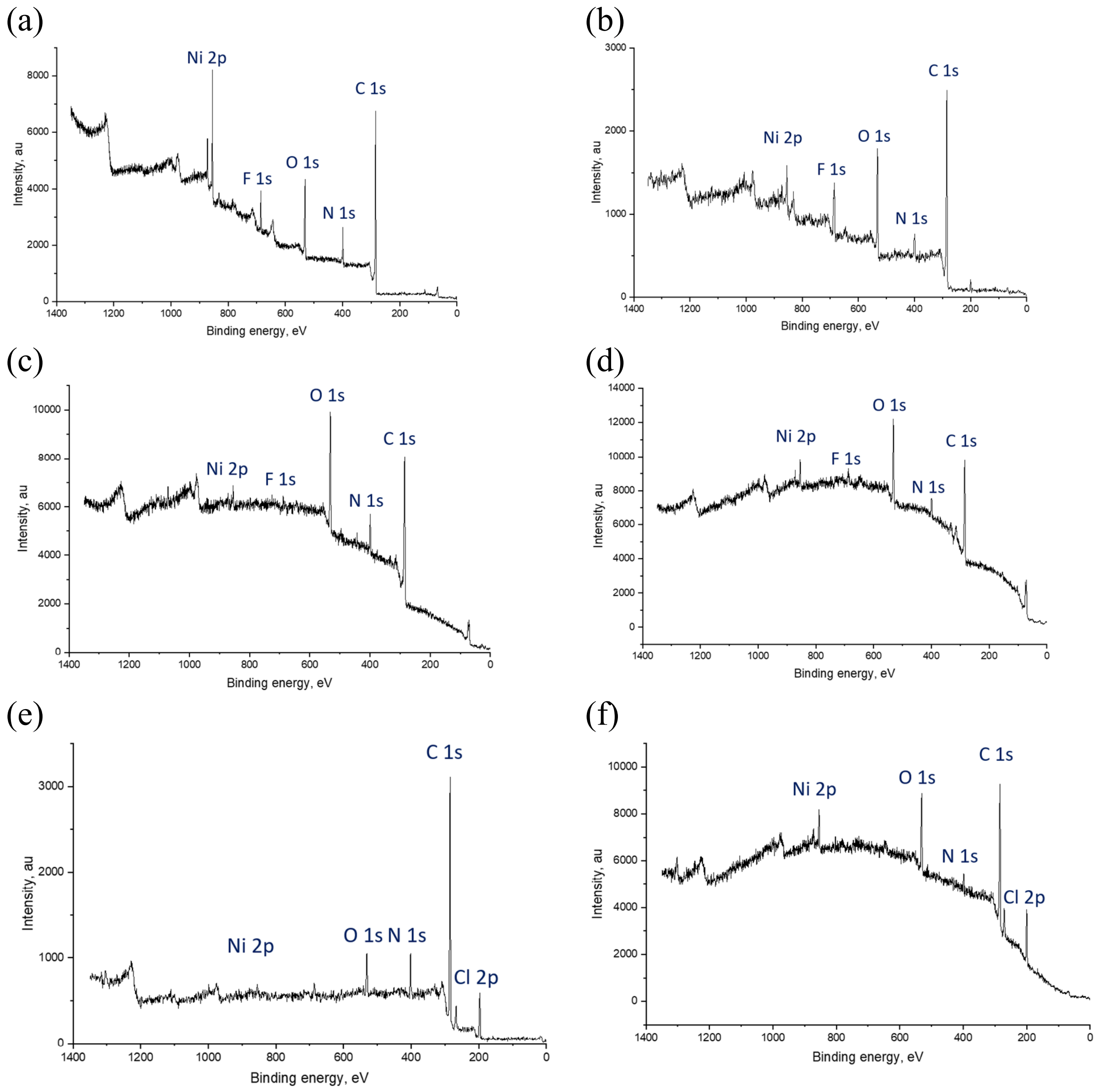

Figure S3. Survey spectra of *poly*[Ni(SalEn)] before (**a**) and after 50 CV cycles in electrolyte with addition of 1 mM Et_4NF (**c**) and 1 mM Et_4NCl (**e**); spectra of *poly*[Ni(SaltmEn)] before (**b**) and after 50 CV cycles in electrolyte with addition of 1 mM Et_4NF (**d**) and 1 mM Et_4NCl (**f**).
